# Alkaline ionic liquids applied in supported ionic liquid catalyst for selective hydrogenation of citral to citronellal

**DOI:** 10.3389/fchem.2014.00003

**Published:** 2014-02-06

**Authors:** Eero Salminen, Pasi Virtanen, Jyri-Pekka Mikkola

**Affiliations:** ^1^Laboratory of Industrial Chemistry and Reaction Engineering, Process Chemistry Centre, Åbo Akademi UniversityÅbo-Turku, Finland; ^2^Laboratory of Technical Chemistry, Department of Chemistry, Chemical-Biological Centre, Umeå UniversityUmeå, Sweden

**Keywords:** catalysis, hydrogenation, ionic liquids, supported ionic liquid catalysts, fine chemicals

## Abstract

The challenge in preparation of ionic liquids containing a strong alkaline anion is to identify a suitable cation which can tolerate the harsh conditions induced by the anion. In this study, a commercial quaternary ammonium compound (quat) benzalkonium [ADBA] (alkyldimethylbenzylammonium) was used as a cation in the synthesis of different alkaline ionic liquids. In fact, the precursor, benzalkonium chloride, is a mixture of alkyldimethylbenzylammonium chlorides of various alkyl chain lengths and is commonly used in the formulation of various antiseptic products. The prepared ionic liquids were utilized as Supported Ionic Liquid Catalysts (SILCAs). Typically, a SILCA contains metal nanoparticles, enzymes, or metal complexes in an ionic liquid layer which is immobilized on a solid carrier material such as an active carbon cloth (ACC). The catalysts were applied in the selective hydrogenation of citral to citronellal which is an important perfumery chemical. Interestingly, 70% molar yield toward citronellal was achieved over a catalyst containing the alkaline ionic liquid benzalkonium methoxide.

## Introduction

Ionic liquids have great potential for developing clean catalytic technologies. Ionic liquids can make a significant positive environmental impact as a replacement for volatile organic solvents. Volatile organic compounds are a major source of environmental pollution. Generally, ionic liquids are molten salts that possess a low melting point and negligible vapor pressure (Bonhote et al., 1996; Rogers and Seddon, 2003; Deetlefs and Seddon, 2006). The two most applied, classical methods for the preparation of ionic liquids are the metathesis procedure and acid-base neutralization reaction. Metathesis implies a reaction of, e.g., a halide salt with a group 1 metal or ammonium salt of the desired anion (Welton, 1996).

Ionic liquids can be divided into acidic, neutral, or alkaline/basic ionic liquids. Acidic and alkaline ionic liquids represent new classes of acids and bases. Various ionic liquid anions can be classified as alkaline. These alkaline anions include e.g., format, acetate, hydroxide, methoxide, butoxide, and the dicyanamide anions. With alkaline ionic liquids, the cation must be stable enough to tolerate the conditions emerging due to the alkaline anion. With alkaline ionic liquids, electrostatic interactions between the cation and anion are stronger than with neutral ionic liquids. The acidity or alkalinity of ionic liquids is governed by the strength of the cation or the anion (Hajipour and Rafiee, 2009).

Citral and its hydrogenation products such as citronellal are widely used in the perfumery and fine chemical industry. Ionic liquids can boost the activity of the supported catalyst in the hydrogenation of alkenes, citral and other α, β-unsaturated aldehydes (Mehnert et al., 2002; Virtanen et al., 2007). Ionic liquids can also stabilize metal nanoparticles and suppress aggregation resulting in enhanced catalytic activities. Citral hydrogenation reactions with palladium catalysts and alkaline promoters such as potassium hydroxide have earlier demonstrated high selectivity toward citronellal (Salminen et al., 2012).It is proposed that the benzalkonium based ionic liquids could take the role of an alkaline promoter, like in the sample case of acetylation reactions of sugars and alcohols with dicyanamide based alkaline ionic liquids (Forsyth et al., 2002). Strong alkaline ionic liquids clearly enhanced the activity of Supported Ionic Liquid Catalysts (SILCA) in the hydrogenation of citral as well as the selectivity toward citronellal. It was also found out that alkaline ionic liquids can take the role of alkaline-modifiers, which was the case when e.g., potassium hydroxide was applied as a promoter leading to a much higher reaction rates and selectivities toward citronellal (Salminen et al., 2012).

## Materials and methods

### Synthesis of ionic liquids

**Figure 1:**
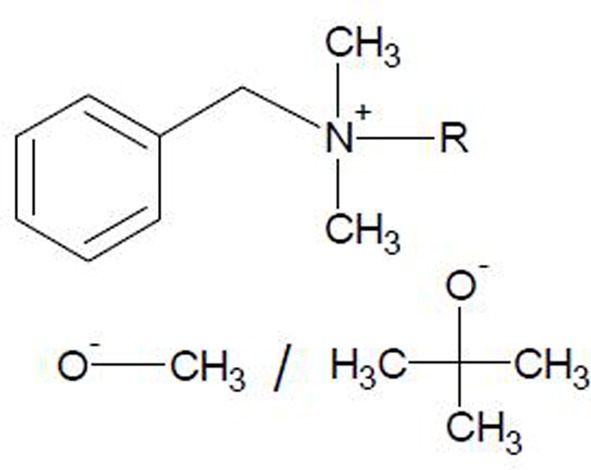
**Synthesized alkaline ionic liquids, benzalkonium methoxide ([ADBA][MeO]) and benzalkonium tert-butoxide ([ADBA][tBuO]).** Alkyl distribution from C_8_H_17_ to C_16_H_33_.

Ionic liquids were prepared by the metathesis procedure as follows: benzalkonium chloride ([ADBA][Cl]) (Acros Organics, 95%) and sodium methoxide (Aldrich, 95%) or potassium tert-butoxide (Acros Organics, 98%), in a molar ratio of 1:1, were dissolved in dichloromethane. The structures of these water miscible ionic liquids are illustrated in Figure 1. It should be noted that neither sodium methoxide nor potassium tert-butoxide are fully soluble in dichloromethane. The solution was stirred at room temperature for 24 h. The precipitated salt and unreacted starting material were filtered off. The ionic liquids dissolved in dichloromethane were washed with a small amount of water. The solvent (dichloromethane) was evaporated under reduced pressure and at an elevated temperature using a rotary evaporator. Consequently, the synthesized alkaline ionic liquids were investigated by means of thermogravimetric analysis (TGA), differential scanning calorimetry (DSC) and NMR-spectroscopy and applied in the preparation of SILCA.

### Characterization of ionic liquids

The alkaline ionic liquids were evaluated for their mutual solubility in different solvents. DSC was applied for analyzing melting points and glass transition temperatures. The decomposition temperatures were analyzed by means of TGA. NMR spectra of the ionic liquids were analyzed applying Bruker AV400 instrument.

### Catalyst preparation

A simple straightforward catalyst preparation method was used (Mikkola et al., 2006). Catalysts were prepared as follows: approximately 150 mg of ionic liquid and 50 mg of palladium acetylacetonate (Aldrich, 99%) were both dissolved into acetone (Merc, p.a). The solutions were mixed together and then poured over a pre-dried active carbon cloth (ACC) Kynol^®^ (approximately 1.2 g). The active carbon cloth containing the precursor and the ionic liquid was dried at 80°C 2 h. Both modified- and unmodified (traditional heterogeneous catalyst consisting of palladium nanoparticles on ACC) SILCAs were pre-reduced prior to the hydrogenation experiments. The catalysts were pretreated in a high-pressure semi-batch reactor (Parr Inc.). Pretreating was performed at 120°C under hydrogen flow of 10 bar for 1 h. Consequently, the catalyst containing palladium nanoparticles in an ionic liquid immobilized on ACC was obtained upon decomposition and reduction of the organic metal salt.

### Catalyst characterization

Catalysts containing alkaline ionic liquids were analyzed by means of energy-filtered transmission electron spectroscopy (EFTEM LEO 912 OMEGA) equipped with an energy dispersive X-ray detector. Prior to the characterization, the samples were crushed and dispersed in n-hexane or in water for the EFTEM analyses. Finally, the samples were dispensed on a specimen holder copper grid (e.g., carbon). The catalysts were analyzed as fresh and as spent ones.

Nitrogen physisorption (Carlo-Erba instruments, sorptometer 1900) was applied to determine the specific surface area and pore volume of the materials. Dubinin's equation was applied to calculate the specific surface area whereas Dollim.-Heal method was used to calculate the micropore volume of the catalysts. Approximately 0.15 g of catalyst (active carbon cloth) was outgassed for 3 h at 120°C to eliminate the moisture. Nitrogen was absorbed and desorbed from the sample material which was subjected to −196°C. The leaching of ionic liquids has been studied in our earlier work and it was discovered that since these ionic liquids are not mutually soluble within hexane, they don't leach from the support to the bulk solvent (Virtanen et al., 2007). The spent catalysts were extracted using methanol to study the accumulation of different hydrogenation decomposition products on the catalyst surface. The extractant solution was analyzed by means of gas chromatography as shown in the case of the citral hydrogenation samples.

### Citral hydrogenation

The SILCA were applied in the hydrogenation of citral (Aldrich, 95%). Experiments were performed in a semi-batch reactor (Parr Instrument Company). The liquid volume of the reaction solvent was 250 ml. The temperature and stirring rate were controlled by a Parr 4843 control unit (Watlow control series 982). The stirring rate was adjusted to 1200 rpm in order to ensure operations under kinetic regime as confirmed by our earlier studies (Virtanen et al., 2007). All experiments were performed at a constant hydrogen pressure and temperature. Approximately 3 g of citral (0.02 mol) was dissolved in 250 ml n-hexane (Merck, >99%).

The samples from the reactor were analyzed by means of gas chromatography (Hewlett Packard 6890 GC with FID). Five hundred microliter of internal standard (0.02 M cyclohexanone in cyclohexane) was added into a 500 μl of sample. The GC column used was an Agilent DB-5 with a length of 60 m, inner diameter of 0.32 mm and a film thickness of 1 μm. The following temperature program was applied: 10 min at 100°C, then raised 5°C/min to 160°C. Temperature was then held 10 min at 160°C. At the end the temperature was increased 13°C/min to 200°C and kept constant for 1 min.

## Results and discussion

### Characterization of ionic liquids

The alkaline ionic liquids were tested for mutual solubility in methanol, acetone, and hexane. The ionic liquids were insoluble (within measuring accuracy) to hexane and soluble to acetone and methanol. The chlorine content of alkaline ionic liquids could not be determined by using AgNO_3_ method because the ionic liquids reacted with AgNO_3_. This was noticed when the same alkaline ionic liquids were synthesized from benzalkonium nitrate and the AgNO_3_ test was positive. As the next step, DSC, a technique for analyzing melting points and glass transition temperatures, was applied. No melting points were found by using DSC (apparatus limit −100°C). The glass transition temperatures were −46°C for [ADBA][tBuO] and −45°C for [ADBA][MeO] salts, respectively. The color of ionic liquids was yellowish/light brown, which implies that some impurities were still present. The decomposition temperatures were analyzed by means of TGA. The decomposition temperatures were 160°C for [ADBA][MeO] and 140°C for [ADBA][tBuO], respectively.

The ^1^H (400 MHz) and ^13^C (400 MHz) NMR spectra of the ionic liquids were analyzed in deuterated chloroform (CDCl_3_). The instrument used was a Bruker AV400. New peaks for the corresponding anion were observed in ^13^C spectrum. ^1^H NMR and ^13^C NMR peaks for [ADBA][tBuO]. ^1^H NMR (CDCl_3_): δ 7.581, 7.334, 4.908 (s, 2H), 3.382 (m, 2H), 3.181 (s, 6H), 1.699 (s, 2H), 1.165 (m, 26H), 0.784 (t, 3H). ^13^C NMR (CDCl3): δ 133.17, 130.45, 129.01, 127.58, 84.10, 73.74, 68.77, 67.34, 63.52, 53.42, 49.56, 31.16, 29.2, 28.45, 26.25, 22.56, 14.00. ^1^H NMR and ^13^C NMR peaks for [ADBA][MeO]. ^1^H NMR (CDCl_3_): δ 7.533, 7.385, 4.771 (s, 2H), 3.381 (m, 2H), 3.127 (s, 6H), 1.748 (s, 2H), 1.234 (t, 20H), 0.832 (t, 3H). ^13^C NMR (CDCl_3_): δ 133.12, 130.65, 129.18, 127.37, 67.82, 63.94, 53.43, 49.45, 31.85, 29.35, 26.3, 22.83, 22.62, 14.06. A new peak for [ADBA][Me] was observed at 53.43 (^13^C NMR spectra for methoxide carbon). New ^13^C NMR peaks for [ADBA][tBuO] were observed at 73.74 and 84.10 (^13^C NMR spectra for t-butoxide anion), respectively.

### Catalyst characterization

**Figure 2:**
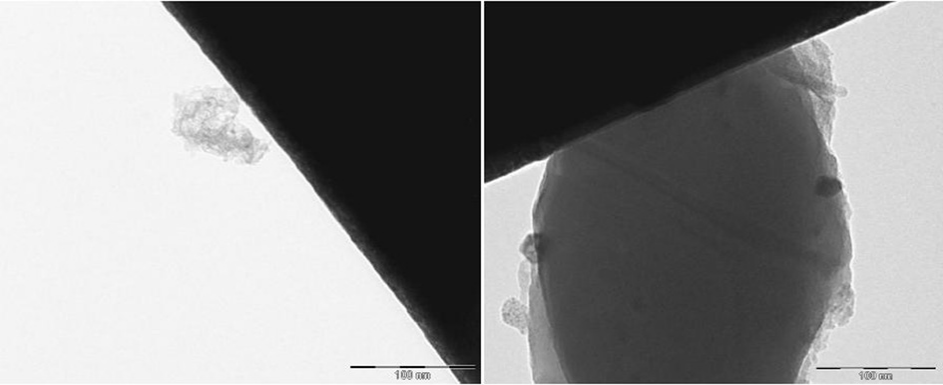
**EFTEM pictures of the catalyst containing palladium in a (ADBA)(MeO) on ACC as fresh (left) and after being exposed to citral hydrogenation reaction**.

**Figure 3:**
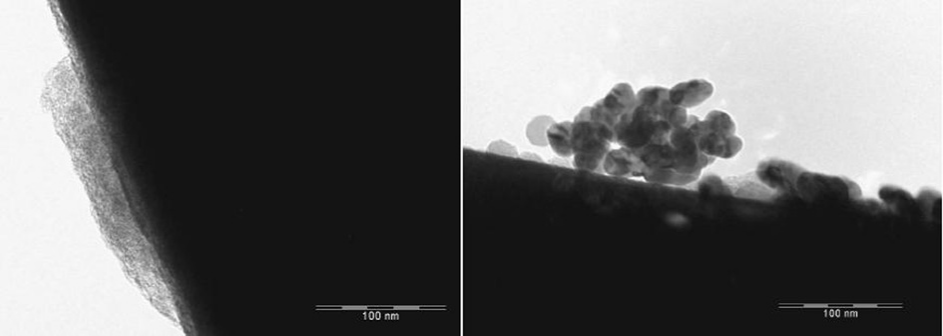
**EFTEM pictures of the catalyst containing palladium in (ADBA)(tBuO)on ACC as fresh (left) and after being exposed to citral hydrogenation procedure**.

The EFTEM images depict the catalysts containing palladium nanoparticles in an ionic liquid layer immobilized on ACC (Figures 2, 3). In a fresh catalyst containing the ionic liquid [ADBA][MeO], the palladium particle size is below 10 nm whereas the particle size of spent catalyst is over 20 nm (Figure 2). Similar agglomeration of palladium particles has been observed with various SILCA catalysts (Virtanen et al., 2007; Salminen et al., 2012). Consequently, this might be one important reason to the deactivation of the catalysts. However, ionic liquids suppress agglomeration. This can be observed when comparing ionic liquid modified catalysts to unmodified catalysts. Palladium particle size can be over 100 nm with the spent Pd/ACC catalyst (Virtanen et al., 2007).

The results from nitrogen physisorption measurements are illustrated in Table 1. The micropore volume of pure ACC was in the range of 0,6 cm^3^/g whereas the specific surface area of pure ACC was 1680 m^2^/g. Smaller surface areas and pore volumes of the spent catalysts with respect to the fresh catalysts indicated that some products and by-products are accumulating into the ionic liquid layer. This is one of the primary reasons for catalyst deactivation. Similar accumulation effects have been observed in the hydroformylation of propene over supported ionic liquid phase catalysts (Riisager et al., 2005). Moreover, accumulation of citral hydrogenation products to catalyst media was observed when the spent catalysts were extracted using methanol.

**Table 1:** **Results from the nitrogen physisorption measurements**.

**Catalyst**		**Surface area (m^2^/g)^a^**	**Micropore volume (cm^3^/g)^b^**
Pd/[ADBA][MeO]/ACC	Fresh	717	0.26
	Spent	106	0.04
Pd/[ADBA][tBuO]/ACC	Fresh	956	0.34
	Spent	82	0.03

a Calculated by Dubinin method.

b Calculated by Dollimore/Heal method.

### Citral hydrogenation

**Figure 4:**
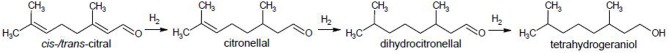
**Citral hydrogenation reaction sequence using alkaline ionic liquids in supported ionic liquid catalysts**.

The hydrogenation of citral was studied using two alkaline ionic liquids immobilized on the catalyst support. These alkaline ionic liquids were synthesized from [ADBA][Cl] and sodium methoxide or potassium tert-butoxide. The catalysts containing alkaline ionic liquids were compared to a catalyst containing [ADBA][Cl] and to a traditional heterogeneous catalyst, consisting of palladium nanoparticles on ACC. Alkaline ionic liquids used in SILCAs were [ADBA][MeO] and [ADBA][tBuO]. The plausible citral hydrogenation reaction scheme is presented in Figure 4.

**Figure 5:**
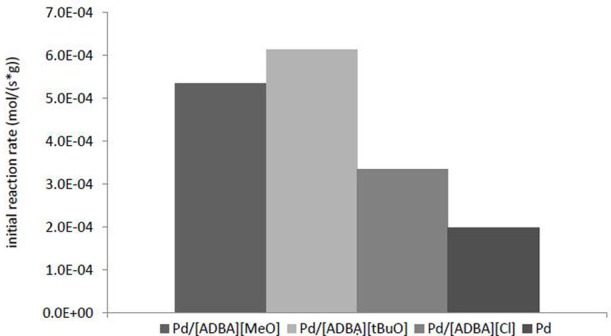
**Comparison of the initial reaction rates with various supported ionic liquid catalysts.** The initial reaction rates were calculated at 5 min from commencing the reaction. All reactions presented here were carried out at *T* = 100°C and *p*(*H*_2_) = 10 bar. The catalysts were Pd in [ADBA][Cl], [ADBA][MeO], and [ADBA][tBuO] on ACC. Pd on ACC is the traditional heterogeneous reference catalyst.

**Figure 6:**
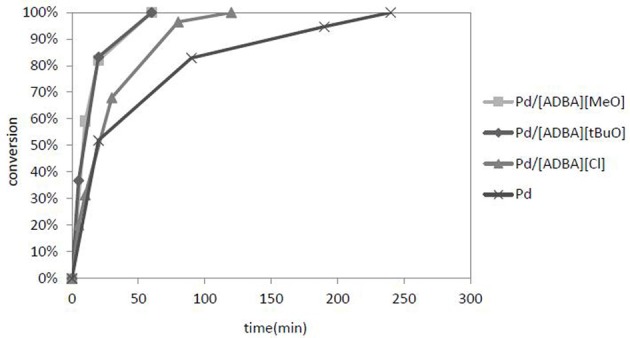
**A graph of citral conversion to products as a function of time.** The catalysts were Pd on ACC and Pd in different ionic liquids on ACC. The ionic liquids were [ADBA][Cl], [ADBA][MeO], and [ADBA][tBuO], respectively. The reaction conditions were *T* = 100°C, *p*(*H*_2_) = 10 bar.

The most active catalyst was Pd in [ADBA][tBuO] on ACC. This supported ionic liquid catalyst was over three times as active as the traditional heterogeneous catalyst (Figure 5). SILCA containing alkaline ionic liquids were the most active catalysts. Moreover, the conversion of citral was faster when using catalysts containing alkaline ionic liquids. The total conversion of citral was reached four times faster with SILCAs containing alkaline ionic liquids than when traditional heterogeneous catalyst (Pd on ACC) was applied (Figure 6).

**Figure 7:**
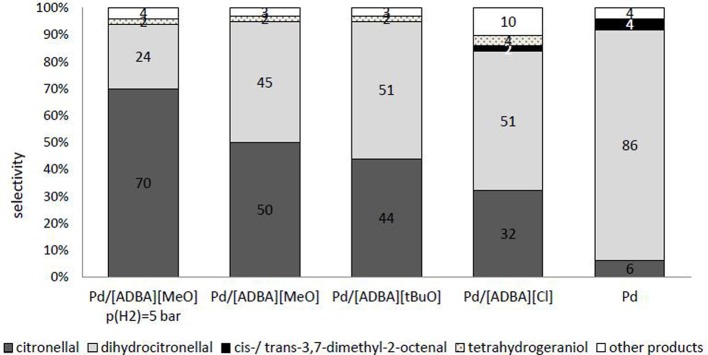
**Comparison of the selectivities of the products from citral hydrogenation over supported ionic liquid catalysts containing an alkaline ionic liquid after complete conversion.** Traditional heterogeneous catalyst (palladium nanoparticles on ACC) and catalyst containing [ADBA][Cl] were chosen as reference catalysts. The reaction conditions were *T* = 100°C, *p*(*H*_2_) = 10 bar. Other products are compounds from cracking and dehydration of citral and its hydrogenation products.

As observed, SILCA consisting of alkaline ionic liquids demonstrated enhanced selectivities toward citronellal. When the catalyst was containing the ionic liquid [ADBA][MeO], the main product obtained was citronellal. When using alkaline ionic liquids in SILCAs, the only hydrogenation products were citronellal, dihydrocitronellal, and tetrahydrogeraniol. Citronellal and dihydrocitronellal were the main products (Figure 7). Other products were compounds from cracking and dehydration of citral and its hydrogenation products. In the first phase, the hydrogenation reaction proceeds almost totally toward citronellal by hydrogenating the conjugated carbon-carbon double bond. From citronellal, the reaction proceeds to dihydrocitronellal and further to tetrahydrogeraniol. Further, in the case of the catalyst containing the alkaline ionic liquid [ADBA][MeO], the molar yield of citronellal increased from 50 to 70% when the hydrogen pressure was lowered from 10 to 5 bar. The increase in citronellal molar yield is predictable, since lowering the hydrogen pressure increases the yield of less hydrogenated, intermediate products (e.g., citronellal). The time required for total conversion increased when pressure was changed from 10 to 5 bar.

Claus et al. demonstrated that the Pd/C catalyst coated with the alkaline dicyanamide based ionic liquid ([C_4_C_1_IM][N(CN)_2_]) is highly selective toward citronellal (Arras et al., 2008). In our experiments (100°C and 20 bar) the activity of the catalyst Pd in 1-butyl-3-methylimidazoliumdicyanamide ([C_4_C_1_IM][N(CN)_2_]) on ACC was very poor, even though the selectivity toward citronellal was high (9% conversion and 90% selectivity in 24 h).

**Figure 8:**
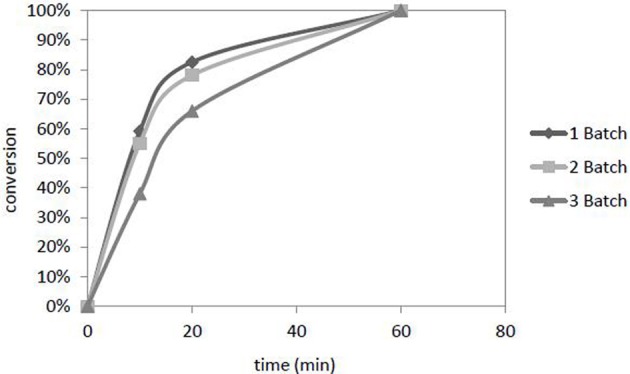
**Deactivation of the catalyst Pd in [ADBA][MeO] on ACC during three consecutive experiments.** Citral conversion to products is presented as a function of time. The reaction conditions were *T* = 100°C, *p*(*H*_2_) = 10 bar.

The catalyst Pd/[ADBA][MeO]/ACC was reused three times in consecutive batches in the transformation of citral (Figure 8). The temperature and pressure were 100°C and 10 bars in every batch, respectively. The plausible reasons for catalyst deactivation are the accumulation of citral hydrogenation products into the ionic liquid layer and agglomeration of palladium particles on the catalyst surface as shown in catalyst characterization section.

With SILCA containing alkaline ionic liquid ([ADBA][MEO]), 70% molar yield of citronellal was obtained whereas alkaline-modified (e.g., KOH) SILCAs, studied in our earlier work, resulted in 74% citronellal molar yields (Salminen et al., 2012). It was observed that SILCAs containing alkaline ionic liquids could be used as a more feasible and catalytically stable alternative to alkaline modified SILCAs. The deactivation of alkaline modified SILCAs is much higher than in SILCAs containing alkaline ionic liquids.

## Concluding remarks

The increase in activity and citral conversion were noticed in SILCAs containing alkaline ionic liquids. Consequently, we propose that these strong alkaline ionic liquids take the role of a basic promoter, which was the case when e.g., potassium hydroxide was applied as a promoter leading to higher reaction rates and selectivities toward citronellal. The most active catalyst, Pd in [ADBA][tBuO] on ACC, was over three times as active as the traditional heterogeneous catalyst. In the case of the SILCA containing the ionic liquid [ADBA][MeO] and upon a hydrogen pressure of 5 bars, the main product was citronellal (yield 70%). Highly selective reaction route toward citronellal was accomplished when applying alkaline ionic liquids in SILCAs.

### Conflict of interest statement

The authors declare that the research was conducted in the absence of any commercial or financial relationships that could be construed as a potential conflict of interest.
